# The effects of an obesogenic diet on behavior and cognition in zebrafish (*Danio rerio*): Trait average, variability, repeatability, and behavioral syndromes

**DOI:** 10.1002/ece3.9511

**Published:** 2022-11-15

**Authors:** Hamza Anwer, Rose E. O'Dea, Dominic Mason, Susanne Zajitschek, Annabell Klinke, Madeleine Reid, Daniel Hesselson, Daniel W. A. Noble, Margaret J. Morris, Malgorzata Lagisz, Shinichi Nakagawa

**Affiliations:** ^1^ Evolution & Ecology Research Centre and School of Biological, Earth and Environmental Sciences University of New South Wales Sydney New South Wales Australia; ^2^ Diabetes and Metabolism Division Garvan Institute of Medical Research Sydney New South Wales Australia; ^3^ Liverpool John Moores University School of Biological and Environmental Sciences Liverpool UK; ^4^ Centenary Institute and Faculty of Medicine and Health University of Sydney Sydney New South Wales Australia; ^5^ Division of Ecology and Evolution, Research School of Biology The Australian National University Canberra Australian Capital Territory Australia; ^6^ Department of Pharmacology, School of Medical Sciences University of New South Wales Sydney New South Wales Australia

**Keywords:** anxiety, cognition, high fat diet, obesogenic diet, personality, variance, zebrafish

## Abstract

The obesity epidemic, largely driven by the accessibility of ultra‐processed high‐energy foods, is one of the most pressing public health challenges of the 21st century. Consequently, there is increasing concern about the impacts of diet‐induced obesity on behavior and cognition. While research on this matter continues, to date, no study has explicitly investigated the effect of obesogenic diet on variance and covariance (correlation) in behavioral traits. Here, we examined how an obesogenic versus control diet impacts means and (co‐)variances of traits associated with body condition, behavior, and cognition in a laboratory population of ~160 adult zebrafish (*Danio rerio*). Overall, an obesogenic diet increased variation in several zebrafish traits. Zebrafish on an obesogenic diet were significantly heavier and displayed higher body weight variability; fasting blood glucose levels were similar between control and treatment zebrafish. During behavioral assays, zebrafish on the obesogenic diet displayed more exploratory behavior and were less reactive to video stimuli with conspecifics during a personality test, but these significant differences were sex‐specific. Zebrafish on an obesogenic diet also displayed repeatable responses in aversive learning tests whereas control zebrafish did not, suggesting an obesogenic diet resulted in more consistent, yet impaired, behavioral responses. Where behavioral syndromes existed (inter‐class correlations between personality traits), they did not differ between obesogenic and control zebrafish groups. By integrating a multifaceted, holistic approach that incorporates components of (co‐)variances, future studies will greatly benefit by quantifying neglected dimensions of obesogenic diets on behavioral changes.

## Graphical abstract

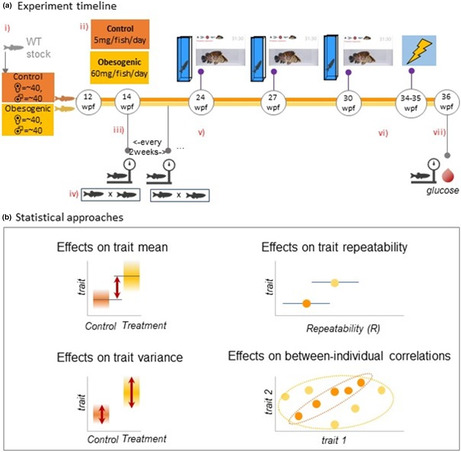

## 1 INTRODUCTION

The obesity epidemic is one of the most pressing public health challenges of the 21st century (Seidell & Halberstadt, 2015). According to World Health Organization (WHO), worldwide obesity has nearly tripled in the last 40–50 years, and numbers are projected to grow at an alarming rate over the next two decades (Finkelstein et al., 2012; World Health Organization, 2017). A major driver of the obesity epidemic is the change to the global food system. Today, ultra‐processed, high‐energy foods are available in large quantities and easily accessible in many countries. This food‐rich environment has resulted in widespread caloric and energy imbalances as well as poor consumer choices which contribute to the development of obesity (Steeves et al., 2014; Zobel et al., 2016). Obesity is typically associated with a cluster of metabolic conditions which increase the risk of cardiovascular disease and diabetes (i.e., metabolic syndrome). Furthermore, there are increasing concerns about its impact on behavior and cognition (Liang et al., 2014; Morris et al., 2015; Romain et al., 2018; Spyridaki et al., 2016; Sutin et al., 2011; Vainik et al., 2019). While studies examining the effects of diet‐induced obesity on behavior and cognition continue to emerge, they are often one‐dimensional, focusing mainly on mean differences between individuals exposed to control and obesogenic diets.

To truly assess treatment efficacy, one must understand how treatments may affect variability in an outcome as well as the mean (Senior et al., 2016). For instance, conventional clinical trials aim to find mean differences and often assume constant effects across subjects. In a review of over 200 clinical trials, however, Cortés et al. (2018) found that one in seven studies had significantly different variances between groups (i.e., heteroskedasticity), leading to a non‐constant effect among individuals. Also, this observation highlights how disregarding variance can lead to a loss of subtle, yet important information. Studies in the medical field have also highlighted the importance of understanding variability to devise treatment strategies that minimize drug resistance and help cater to different populations (e.g., older populations; Frank & Rosner, 2012; McLachlan et al., 2009). The latter has been explored (Goetz & Schork, 2018). The importance of variance has also been argued for in ecology, especially considering that natural selection processes act on variation. For instance, researchers have highlighted how understanding intraspecific differences in trait variation can provide unique insights into the forces structuring communities especially as phenotypic variability plays an important role in promoting diversity and stability in communities (Bolnick et al., 2011; Lang et al., 2021; Maynard et al., 2019; Violle et al., 2012). Perhaps the largest strides have been made in the field of animal behavior over the past two decades, where researchers have incorporated the statistical investigation of variance components to better understand the nuances associated with behavioral traits between individuals, leading to an entire new area of study, ‘animal personality’ (Bell, 2007; Carter et al., 2013; O'Dea et al., 2020; Réale et al., 2007; Roche et al., 2016; Sih et al., 2004).

Animal personality can be defined as the consistent individual differences in behavior across time and contexts (Dingemanse et al., 2010). Animal personality studies measure two properties of (a suite of) behaviors: (i) consistent individual differences over time and space or statistically significant between‐individual variance of behavioral traits, often quantified by intra‐class correlation (also known as repeatability; Bell et al., 2009; Nakagawa & Schielzeth, 2010) and (ii) statistically significant covariance (correlation) among a suite of repeatable behaviors, also known as behavioral syndromes (Dingemanse et al., 2012; Sih et al., 2004). These two measurements (i.e., repeatability and correlations among traits) can be applied in a multi‐faceted, holistic manner to answer the questions related to behavior beyond the field of animal behavior. For instance, this approach could be applied in human and rodent studies examining the effects of a high‐fat diet on behavior and cognition (see review by Freeman et al., 2014), which currently fail to investigate effects on aspects of trait variance, even though meta‐analyses have shown behavioral and cognitive traits are repeatable (Bell et al., 2009; Cauchoix et al., 2018). Taken together, there exists a clear gap as to whether a particular intervention or exposure could trigger changes in variance or correlations among a suite of traits in biomedical research. Zebrafish (*Danio rerio*), a popular model organism in both biomedicine and animal behavior offer the opportunity to explore this notion in depth.

The zebrafish has been widely used as an animal model to answer questions in behavioral neuroscience as well as obesity (Aoki et al., 2015; Zang et al., 2018). As zebrafish possess similar physical characteristics (e.g., body organs for metabolic activities) and pathophysiological pathways as mammals, they respond similarly to obesogenic diets which range from custom high‐fat diets to overfeeding (e.g., increased weight and abnormal levels of triglycerides; Meguro et al., 2019; Oka et al., 2010; Schlegel & Stainier, 2007; Vargas & Vásquez, 2017). Previous studies have shown obesogenic diets resulting in physical as well physiological impairments in zebrafish such as increased body fat, BMI, and adipose tissue; as well as fatty liver disease and high blood glucose (Landgraf et al., 2017; Meguro et al., 2019; Oka et al., 2010; Schlegel & Stainier, 2006; Tainaka et al., 2011; Tingaud‐Sequeira et al., 2011; Vargas & Vásquez, 2017; Zhou et al., 2015). Zebrafish are also a highly social species and have sophisticated sensory and motor systems, making them valuable for assessing social behaviors and cognition (Blaser & Vira, 2014; Green et al., 2012; Pather & Gerlai, 2009; Sison & Gerlai, 2010; Spence et al., 2008). Most notably, zebrafish show robust evidence of personality as well as correlated behaviors (Anwer et al., 2021; Baker et al., 2018; Fangmeier et al., 2018; Moretz et al., 2007; Roy & Bhat, 2018; Thomson et al., 2020). For example, zebrafish display consistent individual differences in behaviors associated with boldness, shyness, and aggression, widely studied personality traits that play important roles in activities such as foraging, reproduction, and survival (Ariyomo et al., 2013; Oswald et al., 2012; Sloan Wilson et al., 1994; Way et al., 2015).

Here, using zebrafish as a model species, we aim to address three questions pertaining to the effects of obesogenic diets on behavioral and cognitive traits. (1) What are the effects of an obesogenic diet on mean and trait variance? (2) Does an obesogenic diet influence trait repeatability (i.e., between‐individual consistency in behavioral traits or “personality”)? (3) Does an obesogenic diet influence correlations among behavioral traits (i.e., behavioral syndromes). To address these questions, we performed a multifaceted experiment including anxiety, personality, and aversive learning measures alongside body weight and fasting blood glucose (FBG). In addition, we investigate sex differences, as they are ubiquitous and there have been repeated calls for the inclusion of sex as an important biological variable in experiments (Zajitschek et al., 2020).

## 2 MATERIALS AND METHODS

### 2.1 Experimental subjects and design

#### 2.1.1 Zebrafish population and husbandry

Zebrafish were derived wild‐type (WT) stock from a mixture of Tübingen long fin, AB and other unidentified strains (which had been interbred for 8–10 generations to increase genetic diversity), and maintained at the Garvan Institute of Medical Research, Sydney, Australia. We housed adult zebrafish in 3.5 L tanks (maximum 24 fish per tank), and larval zebrafish until 1 month of age in 1.1 L tanks (max 50 larval zebrafish per tank). All tanks received recirculating water (pH 7–8, conductivity 500–2500 μS) in a Tecniplast Zebtec System at 28°C under a 12‐h light: 12‐h dark cycle. Zebrafish larvae were fed a standard facility diet of *Paramecium* (twice a day) until 10–12 dpf (days post‐fertilization) at which point they were weaned onto live *Artemia* (twice a day) and dried fish food (once a day). Adult zebrafish were regularly bred to maintain overall health and prevent females developing a plug of clogged eggs which effectively block the oviduct (otherwise known as becoming “eggbound”; Nasiadka & Clark, 2012). All animal experiments were approved by the Garvan Animal Ethics Committee (approval code: ARA 18_18); handling and maintenance followed established protocols.

#### 2.1.2 Experimental cohort

**FIGURE 1:**
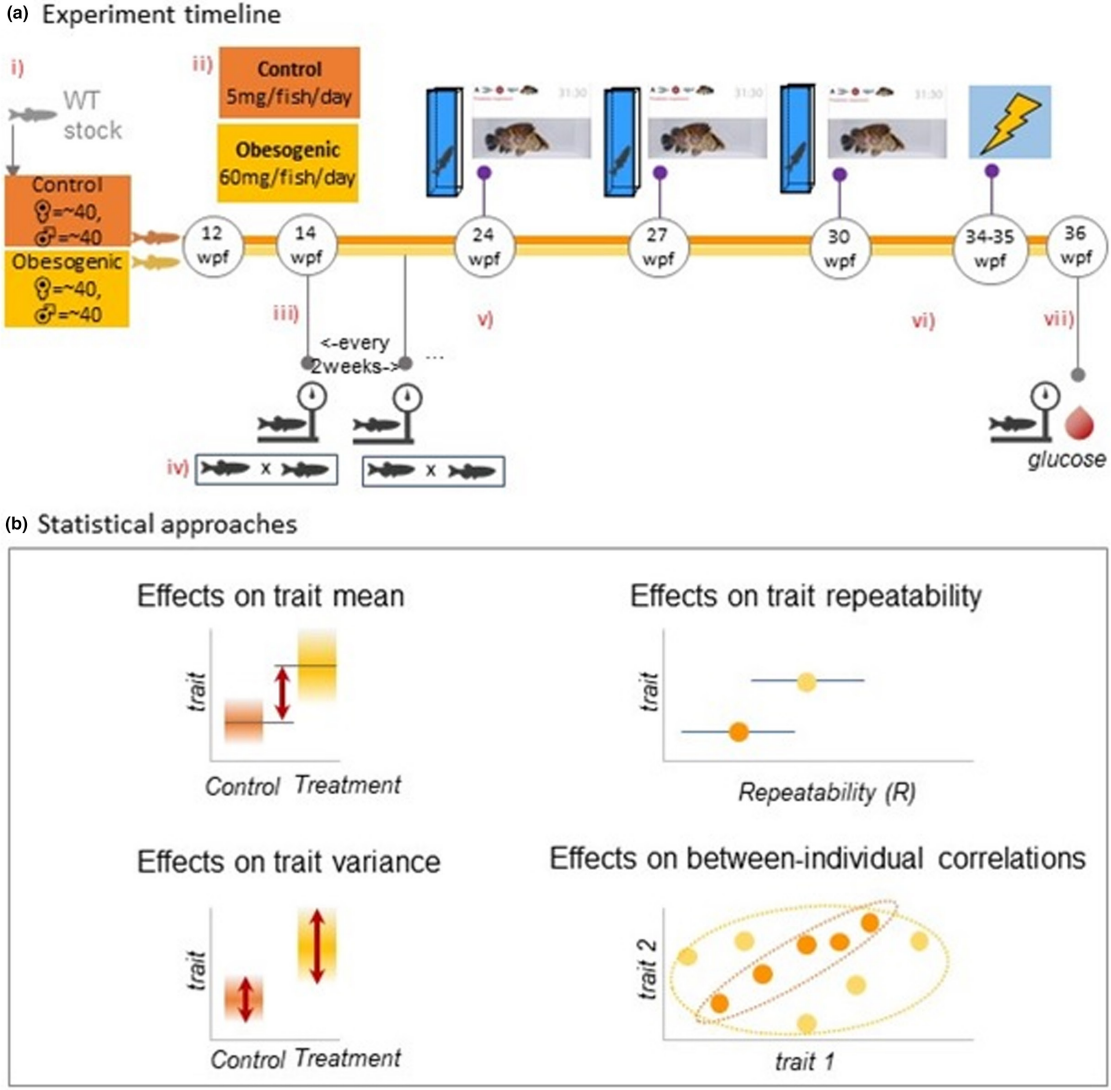
Experimental overview and statistical approach: (a): (i) WT stock assigned to obesogenic and control groups at 12 weeks post‐fertilization (wpf); (ii) designated feeding amounts of Artemia; (iii) body weight measurements began at 14 wpf (continued every 2 weeks until the end of the experiment at 36 wpf); (iv) maintenance breeding within tanks occurred every 2–3 weeks for general health purposes and to prevent females from becoming eggbound (v) behavioral assays began at 24 wpf (3‐week intervals); (vi) aversive learning assay began; (vii) final body weight measurement and fish were sacrificed for FBG (fasting blood glucose) measurements; (b) Statistical approaches: mean and variance differences calculated between control and obesogenic diet zebrafish groups through the use of mixed models; repeatability of behavior estimates calculated as the proportion of between‐group (between‐individual) variance out of total variance; and estimating whether different personality traits correlate at the between‐individual level of variation (known as “behavioral syndromes”).

After 8 weeks post‐fertilization (wpf), we marked zebrafish from 24 independent families with Visible Implant Elastomer tags (VIE, Northwest Marine Technologies, Inc.) for individual identification. We used nine colored tags: red, brown, purple, black, white, yellow, orange, pink, green; and ‘blank’ (no marking). We injected fish tags once on either side of the dorsal fin (Hohn & Petrie‐Hanson, 2013), unless they were designated blanks (hence, no injection). It is important to note, zebrafish were given sufficient time to recover from the trauma of marking as diet manipulation and experiments did not begin for another 4 weeks. We pseudo‐randomly allocated marked fish to experimental and control tanks (4 main tanks per group each with 2 spare tanks; total of 6 tanks per group; 24 fish per tank; zebrafish were housed in 3.5 L tanks), balancing sex ratio and family representation within each tank for statistical independence (Figure 1).

#### 2.1.3 Experimental and control diets

At 12 wpf, adult parental zebrafish (F0) were assigned to either obesogenic (overfeeding) or control diets. Diets were adapted from Oka et al. (2010) and were a method of overfeeding due to its simplicity in producing an obese phenotype (Zang et al., 2018). The diet consisted of freshly hatched Artemia, dried decapsulated Artemia (INVE Artemia Shell Free: An Artemia Nauplii Alternative) and commercially available fish food (O.range GROW‐L). We fed both groups Artemia twice daily (the first feed freshly hatched artemia and the second feed dried artemia): zebrafish in the obesogenic group received 60 mg/fish/day (i.e., 1440 mg/tank equating to 720 mg per feed), while zebrafish in the control group received 5 mg/fish/day (i.e., 120 mg/tank equating to 60 mg per feed). We provided all obesogenic and control tanks with 200 mg of fish food once in the morning to assist with macronutrient requirements. Tanks with excess build‐up of food were cleaned every 2–3 days.

### 2.2 Behavioral assays and other measurements

Behavioral tests began at 24 wpf (at which point zebrafish would have reached adulthood; Figure 1). Behavioral tests were carried out three times (at 3‐week intervals). Due to competitive hierarchies in relation to food access among zebrafish in tanks (Paull et al., 2010), we used 20 fish from each main tank (*n* = 80 control, *n* = 80 treatment; total 160; Figure 1) excluding two of the heaviest males and two of the heaviest females from control tanks (likely four most dominant individuals), and two of the lightest males and two of the lightest females from the treatment tanks (likely four most subordinate individuals). Zebrafish that died were replaced with a counterpart from a spare tank. Each set of trials was conducted within 1 week, followed by a 2‐week recovery gap. Thereafter, we subjected fish to aversive learning tests for 1 week (repeated twice over a 2‐week period). We pseudorandomized both control and treatment tanks to account for day of experiment as well as time of day. We randomly selected Individuals to run in trials. We sacrificed zebrafish at 36 wpf (a sufficient time point to ensure a treatment effect had been induced) for measurements of FBG.

#### 2.2.1 Anxiety assay

We followed the procedure we developed earlier, as described in Anwer et al. (2021). This method uses a taller tank than traditional apparatus. Our work has shown that this type of tank generates more between‐individual differences and is suited for detecting subtle differences in behavior. Based on this finding, we focused our analysis on two highly repeatable, less correlated behaviors: (1) time spent in the low zone (s) and (2) total distance traveled (cm). As each anxiety assay was approximately 8 min long, trials began at 10 a.m. and ended at 4 p.m. This ensured that for each of the three assay sessions, we tested all fish in a single day (one zebrafish was allocated per testing tank of which there was six, which were filmed simultaneously). Water changes occurred every hour to minimize decreases in temperature (water was maintained at ~28°C) and the effects of stress hormones from fish already trialed (Pavlidis et al., 2013).

#### 2.2.2 Personality assay

A video stimulus approach was used to elicit behavioral responses and quantify personality, as described in (Fangmeier et al., 2018). Videos were 38 min in length and comprised of a 34‐min stimulus period, surrounded by two 2‐min buffer periods consisting of blank images. Blank images were still frames of the inside of the tanks used to film the stimulus videos. All buffer periods displayed blank images. The stimulus period consisted of 6 phases. Phases 1 and 6, the “exploration” phases, consisted of a 3‐min blank image (there is a brief buffer period before Phase 6). Phases 2–5, the “stimulus” phases, consisted of a buffer period followed by a 3‐min stimulus period (see Table 1). The stimulus videos (as phases) were relevant to five personality traits: (1) exploration (movement in a novel environment), (2) boldness (in response to exposure of an animated predator model), (3) neophilia (in response to exposure of an animated novel object), (4) aggression (in response to a single aggressive conspecific), and (5) sociability (in response to a shoal of conspecifics). To quantify behavior, we measured total distance traveled during the exploration phase, and time spent near the stimulus screen for the remaining phases.

**TABLE 1:** A timeline of tasks digitally presented to zebrafish with information on what was digitally displayed during each phase.

Task	Pre‐Buffer	Phase 1	Phase 2	Phase 3	Phase 4	Phase 5	Phase 6	Post‐buffer	Total
		Exploration	Boldness	Neophobia	Aggression	Sociability	Exploration		
Buffer (min)	2		3	4	4	4	1	2	
Stimulus (min)		3	3	3	3	3	3		38
Display	Blank	Blank	Predator	Novel object	Single aggressive conspecific	Shoal of conspecifics	Blank	Blank	

#### 2.2.3 Aversive learning assay

We used aversive learning (Pavlovian fear conditioning) to assess cognitive ability in zebrafish fed obesogenic and control diets. Behavioral tests were performed using the Zantiks [AD] fully automated behavioral testing boxes (Zantiks Ltd.). Our protocol for aversive learning (using color as stimuli) followed a previous study in our lab by Mason et al. (2021), which involved exposing zebrafish to stimuli from the base of the Zantiks unit (see Appendix S1 for more details). We quantified learning as the difference in time spent in the CS+ (the color associated with the negative stimulus, a mild electric shock) before and after the aversive experience. A higher difference value indicates less time spent in the CS+ following the aversive experience. Differences are standardized to seconds per minute.

#### 2.2.4 Measurements of body weight and fasting blood glucose

Body weight (g) measurements for F0 were taken at 12 wpf and continued fortnightly, using an AND EJ‐123 scale (Figure 1). At the end of the study, experimental fish were anesthetized in tricaine (4.2 ml of 0.4% in 100 ml system water) for 30 s before decapitation to allow for blood measurement of glucose levels. Glucose levels (mmol/L) were analyzed using glucose meters (Freestyle Freedom Lite).

### 2.3 Statistical analysis

We conducted all statistical analyses in the R environment (Version 3.4.3; R Core Team, 2021) with R Studio (Version 1.1.453; RStudio Team, 2021); all R code used in this study is available at: (https://github.com/Apex619/F0_Chapter_Analysis). For all behavioral assays, we compared mean and variance differences between groups and obtained repeatability estimates of the aforementioned traits. In addition, we estimated between‐individual correlations between traits in our personality assay using a Bayesian approach because we could not obtain errors for between‐individual correlations by using likelihood‐based approaches.

The residual normality of all the response variables was visually checked for all behavioral parameters and transformed for the following variables: personality (time spent near the stimulus screen was square‐root transformed for the predator and novel phase; the transformation was not required for the predator phase in the Bayesian analysis). In all models, we used experimental group (i.e., our experimental condition) and sex as interacting fixed factors (except for Bayesian models in which data were subsetted by experimental condition, therefore only requiring sex as a fixed factor). In anxiety analyses we added water condition as a scaled additional fixed effect (a temporal factor to control for fish being trialed in water that had not yet been changed and therefore exposed to stress hormones from other fish). We used fish ID as a random (clustering) factor in all models.

#### 2.3.1 Mean and variance differences

To calculate mean and variance differences in the aforementioned traits between groups, we used linear mixed models implemented in the function lme in the nlme package (version 3.1‐148; Pinheiro et al., 2020). This function allowed us to model different residual variances between two groups. To model different residual variance between control and treatment groups, we specified the weight argument in the lme function. We also ran the same models assuming a constant variance between the two types of tanks. The two models were compared by likelihood ratio tests using the anova function from the R “stats” package (version 3.6.2) to examine statistical significance when modeling different variances.

#### 2.3.2 Repeatability

Repeatability is defined as the proportion of between‐group (between‐individual) variance out of total variance (Sokal & Rohlf, 2012). To calculate repeatability estimates between control and treatment groups, and then between males and females in control and treatment groups, we used rptR (Version 0.9.21; Stoffel et al., 2017), a package based on a mixed‐effects model framework using the R package lme4 (version 20; Bates et al., 2014). All estimates were “unadjusted” repeatabilities (Nakagawa & Schielzeth, 2010), and only included individual fish IDs as a random effect (with the exception for anxiety estimates which included water condition as a fixed factor).

We obtained standard error and 95% confidence intervals (CIs) using rptr, which employs parametric bootstrapping (Faraway, 2016) with all models set to have 10,000 bootstrap samples. Repeatability estimates with CIs not overlapping 0 were considered statistically significant. In addition, we calculated contrasts between repeatability estimates. We achieved this by calculating the differences between estimated bootstrap distributions and obtaining quantiles at 2.5% and 97.5% from the difference. Contrasts (subtracting a distribution with a higher mean from that with a lower mean) were deemed significant if the difference distribution did not fall below the 2.5% threshold.

#### 2.3.3 Estimating between‐individual correlations

We performed separate multivariate analyses to estimate whether different personality traits correlated at the between‐individual level of variation (known as “behavioral syndrome”), and whether the strength of such correlation differed between control and obesogenic diet groups. To estimate between‐individual correlations, bivariate models were fitted for all combinations of the response variables (that is, all pairs of exploration, aggression, boldness, neophilia, and sociability measurements, resulting in 10 models for the overall analysis of the entire dataset, and 10 models each for control and treatment groups in the analysis of the subsets).

All multivariate mixed models were fitted with a Bayesian approach using the brms package (Bürkner, 2017). We set all models to four chains, each with 6000 iterations and a warm‐up of 2000 iterations. To explore whether the obesogenic treatment affected the presence of behavioral syndromes, we compared posterior distributions of the between‐individual correlations estimated for each pair of behavioral traits.

## 3 RESULTS

### 3.1 Body weight and fasting blood glucose

**FIGURE 2:**
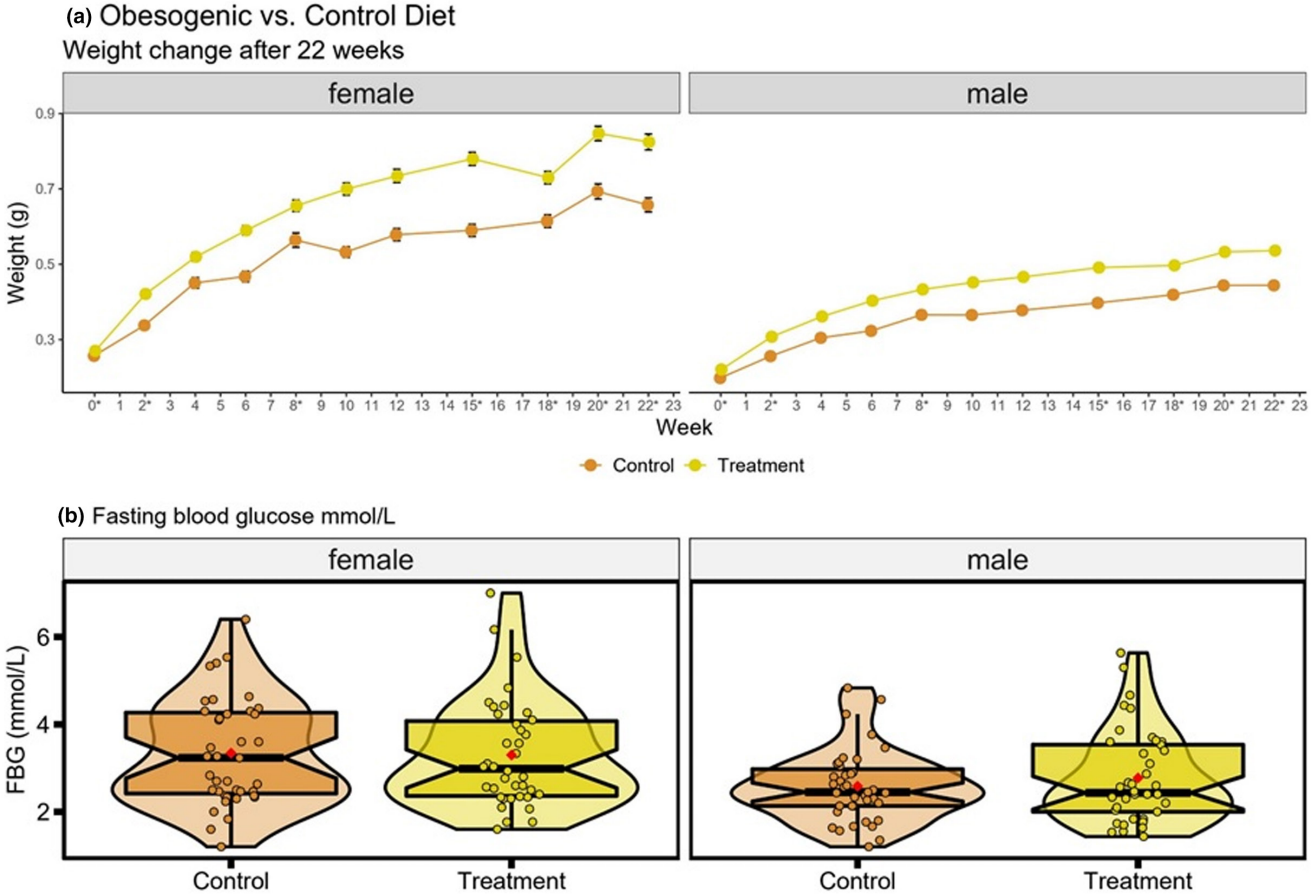
Body weight and fasting blood glucose (a) Body weight changes over a period of 22 weeks from first exposure for both males (*n* = 39–42) and females (*n* = 37–42) in control and obesogenic treatment groups (weeks marked with an asterisk are those where zebrafish were group‐bred for health maintenance purposes). Data shown are mean ± SEM; and (b) Distributions of fasting blood glucose (mmol/L) at the end of the experiment, for control and obesogenic treatment group zebrafish, by sex. Circles represent mean value of three measures taken for each individual (males: *n* = 39 control, *n* = 41 obesogenic group, females: *n* = 39 control, *n* = 38 obesogenic group). Box plots show the median, 95% confidence interval, quantiles, and outliers. Violin plots display the distribution density. Average of mean values are denoted by red diamonds.

Overall, male, and female zebrafish fed the obesogenic diet were significantly heavier than their control counterparts after 22 weeks of diet exposure (*treatment female – control female est* = 0.13, *df* = 171, *t* = 9.28, *p* < .0001; *treatment male – control male est* = 0.06, *df* = 171, *t* = 4.32, *p* = .0002; see Figure 2a). Female zebrafish were heavier than males in both groups (*treatment female – treatment male est* = 0.21, *df* = 171, *t* = 14.81, *p* < .0001; *control female – control male est* = 0.14, *df* = 1583, *t* = 10.46, *p* < .0001) and responded to the obesogenic diet with a greater increase in weight than male zebrafish (*treatment female – treatment male est* = 0.13. *df* = 171, *t* = 9.28, *p* < .0001; Group[treatment]*Sex[male] est = −0.07, *df* = 171, *t* = −3.60, *p* < .0001). On average, for every fortnight of the experiment, zebrafish gained 0.02 g (*df* = 1583, *t* = 61.01, *p* < .0001). In addition, zebrafish on the obesogenic diet displayed significantly more variation in body weight than control zebrafish (25%, *p* < .0001).

There were no significant differences between obesogenic and control zebrafish in FBG levels (Figure 2b; Table S1). However, males had significantly lower FBG than females (LMM contrast *male est* = −0.76, *df* = 153, *t* = −3.06, *p* = .003). Both sexes displayed no significant difference in variance (Figure 2b; Table S1).

### 3.2 Behavioral traits: comparing means and variances

#### 3.2.1 Anxiety behaviors

**FIGURE 3:**
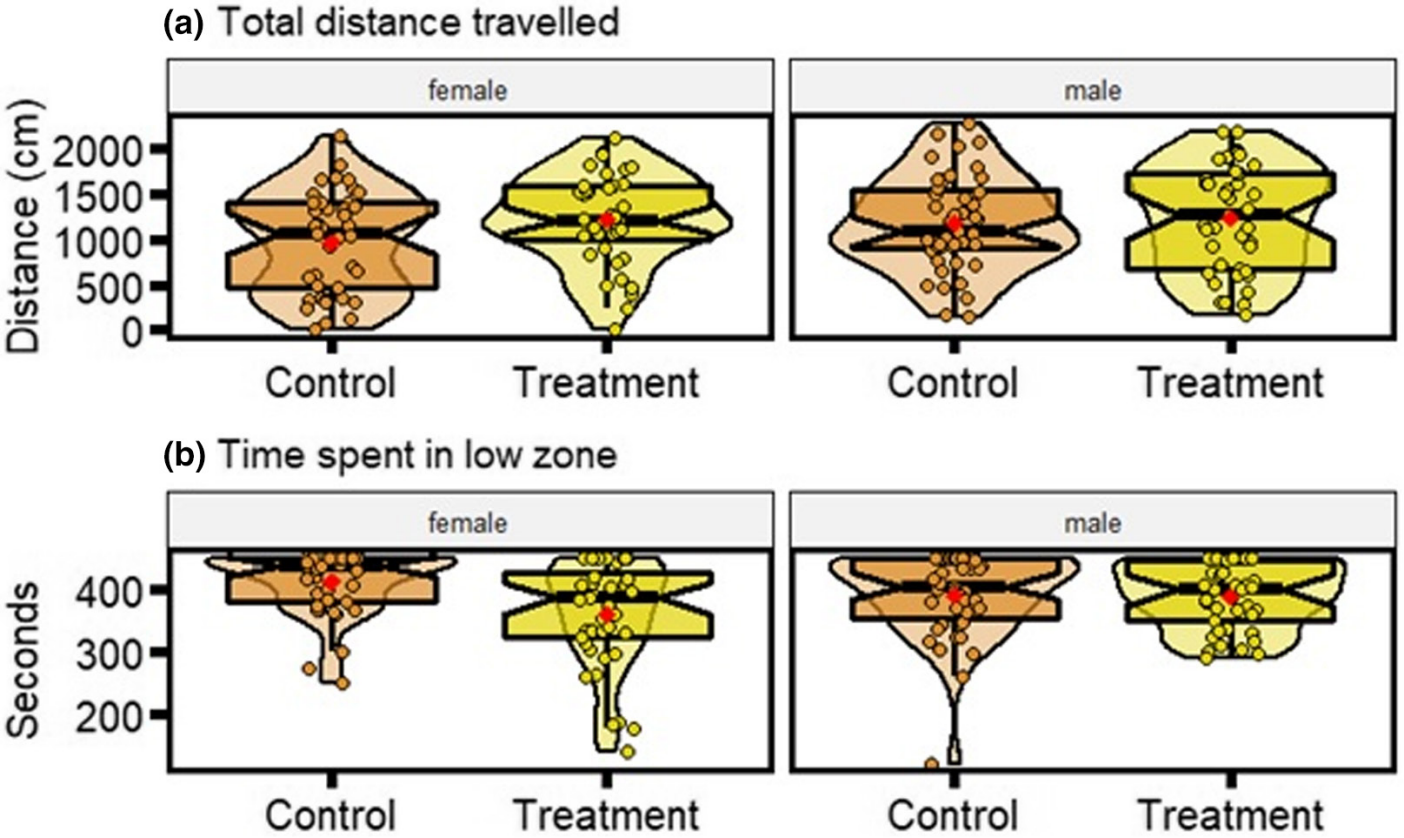
Distributions of behavioral parameters for anxiety assay (a) total distance traveled (cm) and (b) time spent in the low zone (seconds). Data displayed is for control and treatment zebrafish, of each sex. Each plot displays mean individual data points for males (*n* = 43 control, *n* = 41 treatment) and females (*n* = 40 control, *n* = 40 treatment) from three observations. Box plots show the median, its 95% confidence interval, quantiles, and outliers. Violin plots display the distribution density. Average of mean values are denoted by red diamonds.

**FIGURE 4:**
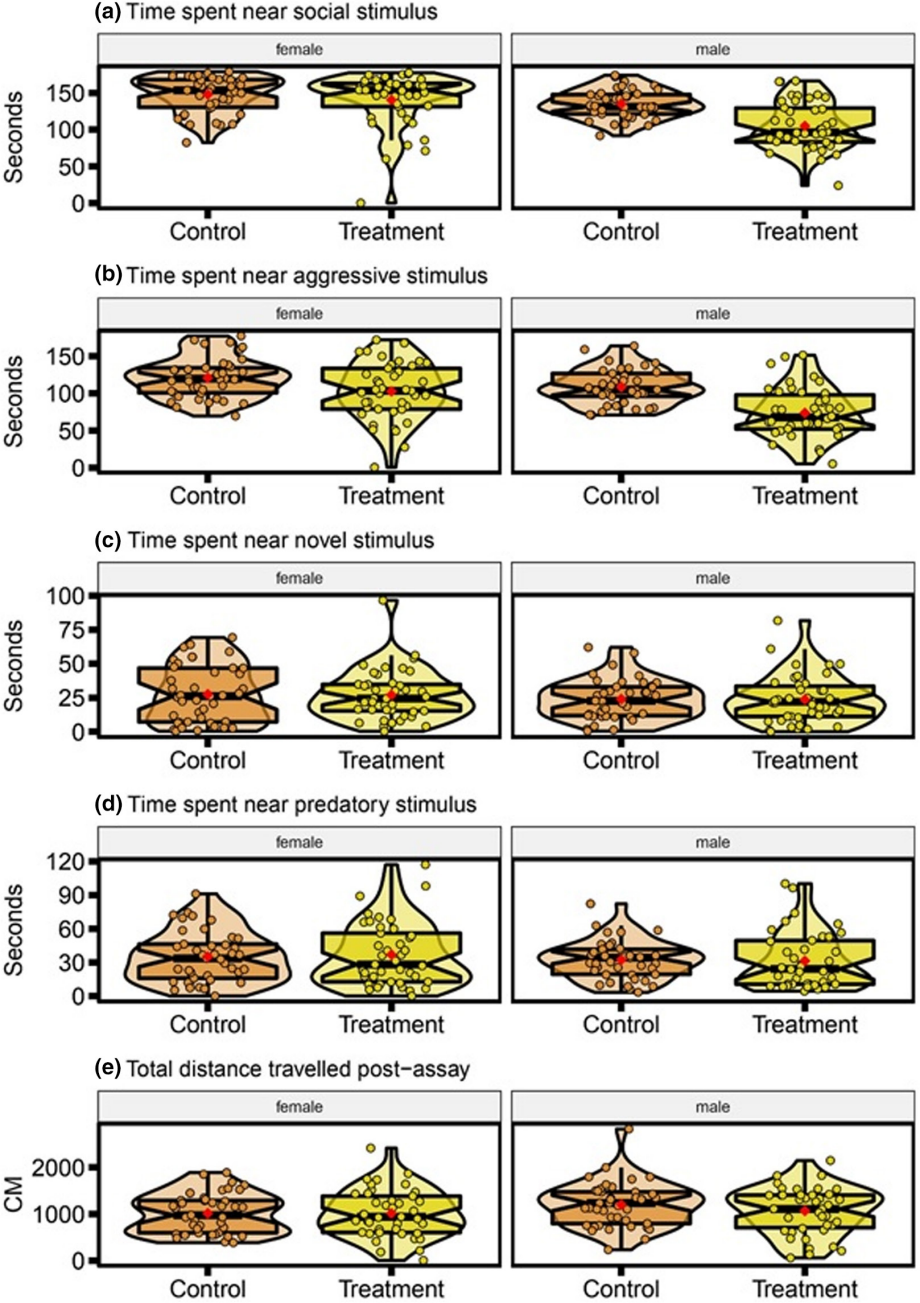
Distributions of behavioral parameters for personality assay (a) time spent near the social stimulus (seconds), (b) time spent near the aggressive stimulus (seconds), (c) time spent near the novel stimulus (seconds), (d) time spent near the predatory stimulus (seconds), (e) total distance traveled during the postassay period (cm). Data displayed as control and obesogenic treatment groups, separated by sex. Each plot displays mean individual data points for males (*n* = 41 control, *n* = 42 obesogenic) and females (*n* = 41 control, *n* = 41 obesogenic) from three observations. Box plots show median, 95% confidence interval, quantiles, and outliers. Violin plots display the distribution density. Average of mean values are denoted by red diamonds.

There were no statistically significant differences between control and obesogenic zebrafish groups in total distance traveled in the tank (Figure 3a; Table S2). However, zebrafish on the obesogenic diet spent significantly less time than control zebrafish in the low zone (LMM contrast *est* = −49.01, *df* = 160, *t* = −3.37, *p* = .001; Figure 4b). Males from the obesogenic group were spending, on average, more time in the low zone than females from the same group (Group[treatment]*Sex[males] est = 50.33, *df* = 160, *t* = 2.48, *p* = .014; Figure 3b). In addition, water condition had no influence on all parameters except for total distance traveled (Table S2). Zebrafish in the obesogenic diet group displayed significantly more variation in the time spent in the low zone (31%, *p* = .0008; Figure 4b). No statistically significant differences in variance were observed between control and obesogenic zebrafish for total distance traveled (*p* = .094; Figure 4a).

#### 3.2.2 Personality traits

**FIGURE 5:**
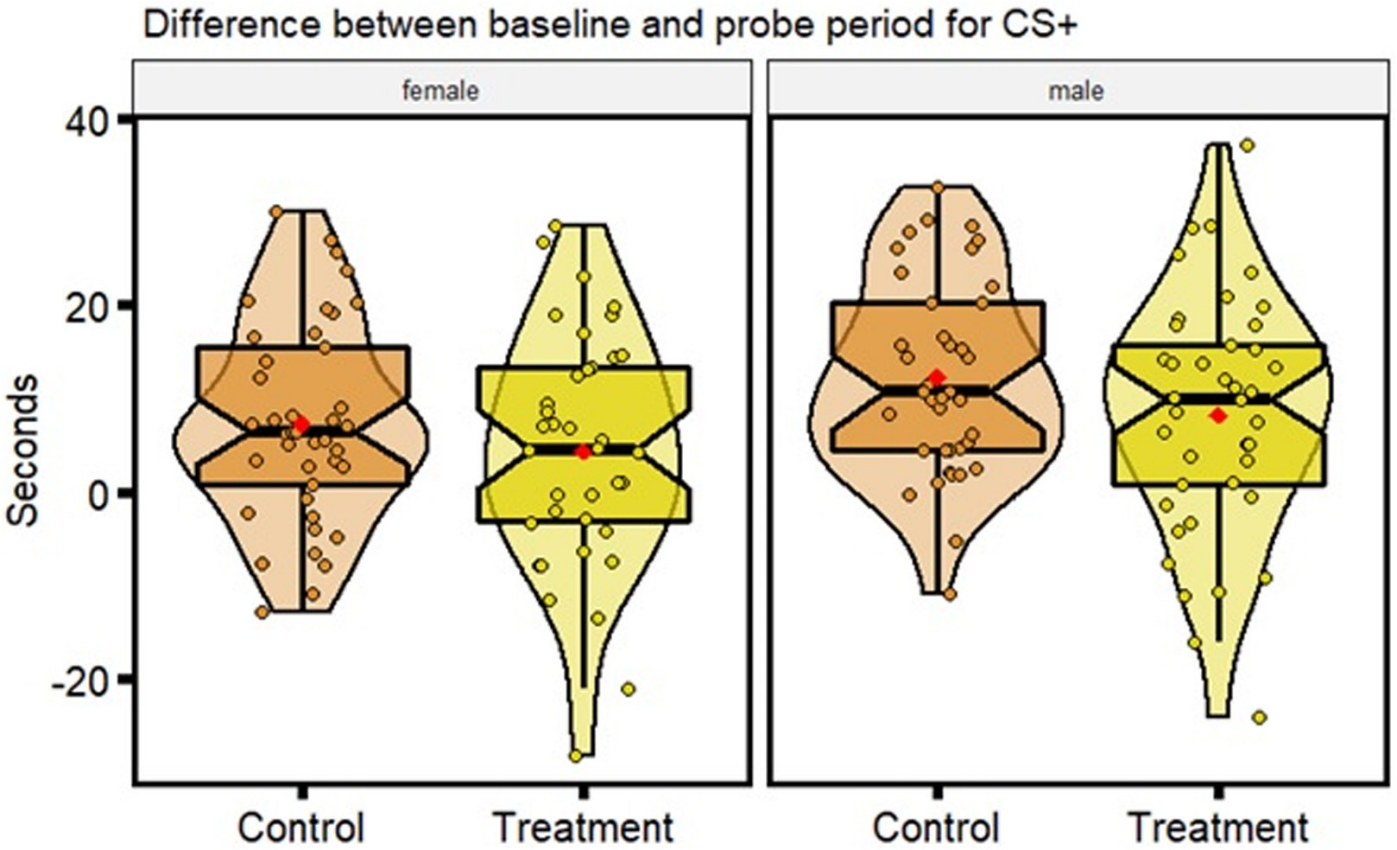
Distributions of behavioral parameters for aversive learning assay: Differences in time spent in the CS+ (color associated with the negative stimulus, a mild electric shock) before and after the aversive experience (seconds), with higher values indicating less time spent in the CS+; data displayed is for control and obesogenic treatment zebrafish, subsetted by sex. Each plot displays mean individual data points for males (*n* = 39 control, *n* = 41 obesogenic) and females (*n* = 41 control, *n* = 38 obesogenic) from two observations. Box plots show the median, 95% confidence interval of the median, quantiles, and outliers. Violin plots display the distribution density. Average of mean values are denoted by red diamonds.

There were no statistically significant differences between control and obesogenic zebrafish in time spent near the stimulus screen during the social (LMM contrast, *treatment est =* −5.35, *df* = 161, *t* = −0.90, *p* = .369; Figure 4a), novel (LMM contrast, *treatment est* = −0.11, *df* = 161, *t* = −0.25, *p* = .804; Figure 4c) or predator phase (LMM contrast, *treatment est* = −0.09, *df* = 161, *t* = −0.18, *p* = .855; Figure 4d); nor in total distance traveled time during the exploration phase (LMM contrast, *treatment est* = −12.39, *df* = 161, *t* = −0.12, *p* = .905; Figure 4e). When faced with the aggression stimulus, obesogenic zebrafish spent significantly less time near the stimulus screen than control zebrafish (LMM contrast, *est* = −17.19, *df* = 161, *t* = −2.56, *p* = .011; Figure 4b). During the social phase, males spent significantly less time near the stimulus screen (LMM contrast, *males est* = −12.80, *df* = 161, *t* = −2.15, *p* = .033; Figure 4a) and more so if they were from the obesogenic treatment group (LMM contrast, Group[treatment]*Sex[males] est = −24.42, *df* = 161, *t* = −2.91, *p* = .004; Figure 5a). Except for total distance traveled during the exploration period, we observed significant differences in variance between control and obesogenic zebrafish in all behavioral phases. Zebrafish fed the obesogenic diet were overall more variable than control zebrafish (social: 33%, *p* < .0001; aggression: 23%, *p* = .008; novel: 22%, *p* = .009; predator 16%, *p* < .04; Figure 4a–d respectively).

#### 3.2.3 Aversive learning

Overall, while zebrafish fed an obesogenic diet displayed a tendency for poorer performance in aversive learning assays, both control and treated groups displayed similar differences between the baseline and probe period for time less spent in the conditioned stimulus (LMM, obesogenic *est* = −2.98, *df* = 155, *t* = −1.13, *p* = .26; Figure 5). In addition, there was no statistically significant difference in variance between control and obesogenic zebrafish (Figure 5).

### 3.3 Behavioral traits: comparing repeatabilities

#### 3.3.1 Anxiety behaviors

**FIGURE 6:**
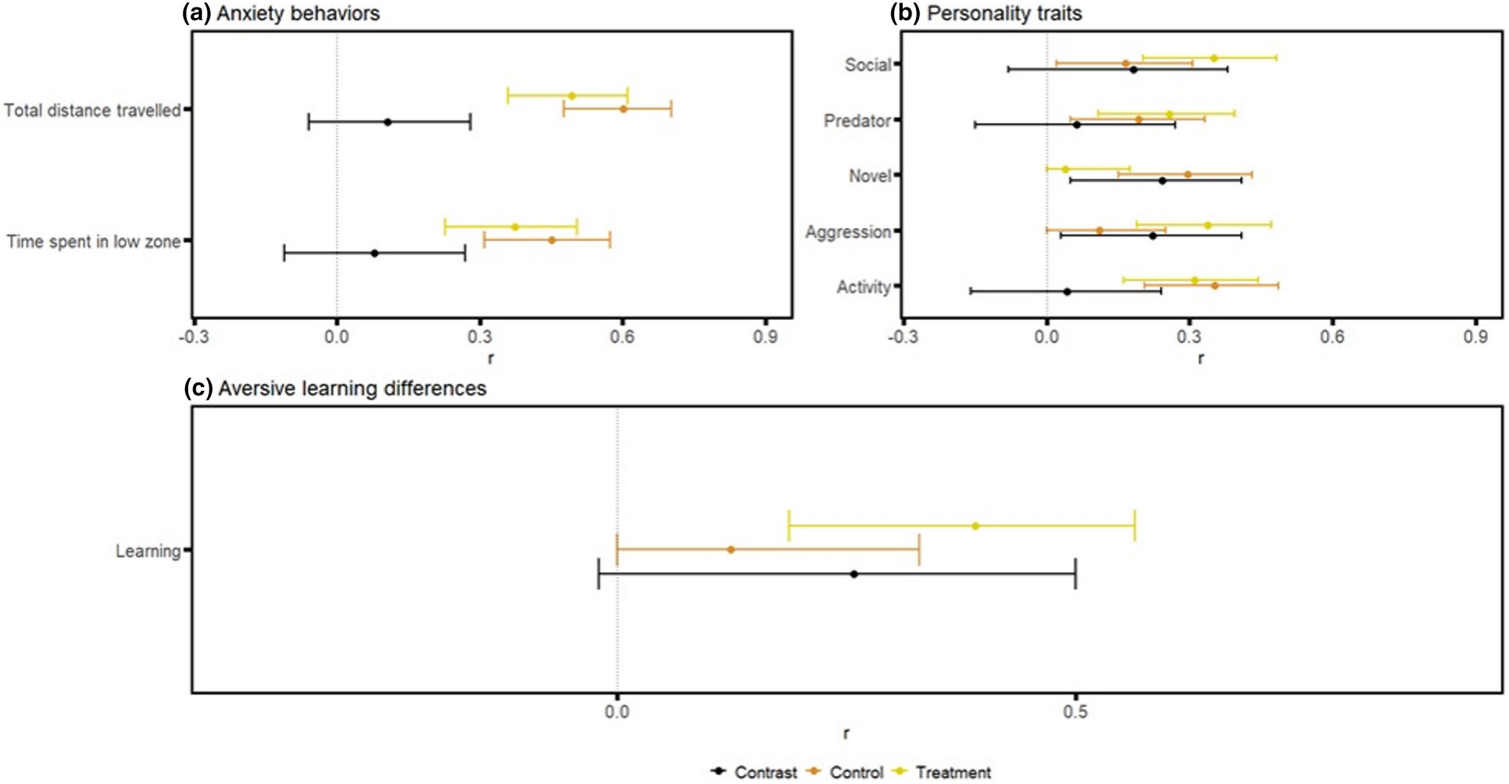
Forest plots of all repeatability estimates: Plot displays repeatability estimates for: (a) anxiety parameters total distance traveled, and time spent in the low zone; (b) personality traits of time spent near the stimulus during the social, predator, novel, and aggression phases, and total distance traveled for the activity phase; and (c) difference in time spent in the CS+ (color associated with the negative stimulus, a mild electric shock) before and after the aversive experience. Repeatability estimates are deemed statistically significant if the associated 95% confidence interval does not cross 0. The contrasts between the control and treatment group are deemed significant if the associated confidence interval does not cross 0.

Both anxiety parameters – total distance traveled, and time spent in the low zone were significantly repeatable for control and obesogenic treatment group zebrafish (total distance traveled: control *R* = 0.60, 95% CI [0.48–0.70], treatment *R* = 0.49, 95% CI [0.36–0.61]; time spent in the low zone: control *R* = 0.45, 95% CI [0.31–0.57], treatment *R* = 0.37, 95% CI [0.23–0.51]; Figure 6a). Although the control group displayed higher repeatability estimates than the obesogenic treatment group for both behavioral parameters, there were no statistically significant differences between the two groups (Figure 6a). Behaviors were also significantly repeatable for both total distance traveled, and time spent in the low zone when analyzed separately in males and females (except for time spent in the low zone for males), although no statistically significant sex differences were detected (Figure S3).

#### 3.3.2 Personality traits

**FIGURE 7:**
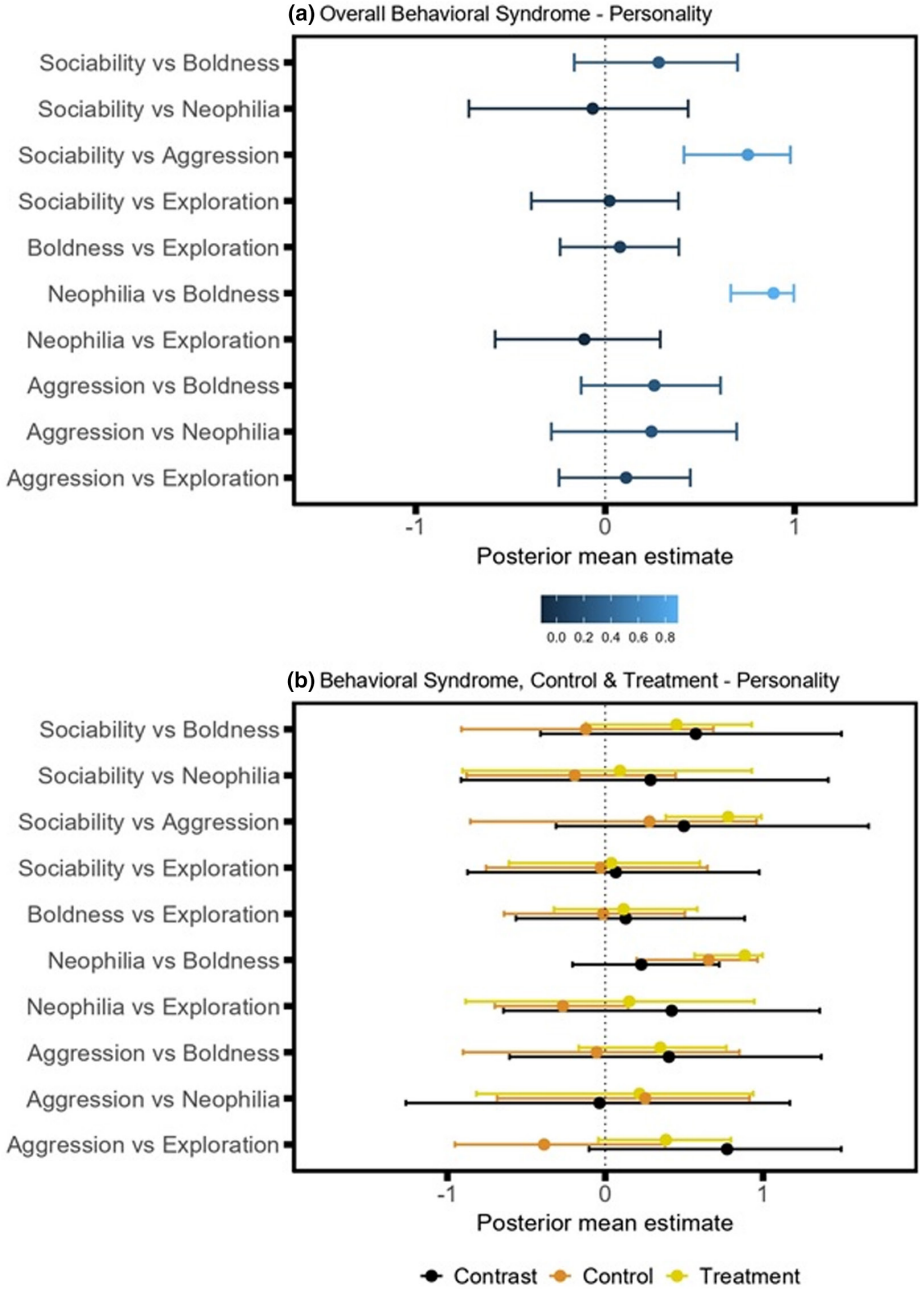
Forest plots of mean posterior estimates from bivariate models: Plot displayed mean and 95% credible intervals for (a) overall behavioral syndrome analysis (lighter blue shades indicate higher posterior mean estimates); and (b) behavioral syndrome analysis subsetted by group, with contrast analysis to determine differences between control and treatment zebrafish. Posterior mean estimates are deemed significant if the associated 95% confidence interval does not cross 0. The contrasts between the control and treatment group are deemed significant if the associated confidence interval does not cross 0.

The parameters total distance traveled (during the exploration phase) and time spent near the stimulus screen (during all other phases) were significantly repeatable in both control (apart from the aggression phase) and obesogenic treatment zebrafish (apart from the novel phase; Figure 6b). Overall, zebrafish from the obesogenic zebrafish treatment group had higher repeatability values during the social phase (Control: *R* = 0.29, 95% CI [0.16–0.44]; Treatment: *R* = 0.30, 95% CI [0.17–0.45]), predator phase (Control: *R* = 0.26, 95% CI [0.11–0.40]; Treatment: *R* = 0.26, 95% CI [0.14–0.40]) and aggression phase (Control: *R* = 0.11, 95% CI [0–0.27]; Treatment: *R* = 0.32, 95% CI [0.19–0.50]). However, repeatabilities were only significantly different between control and obesogenic zebrafish during the aggression phase (Contrast: 95% CI [0.01–0.26]; Figure 7b) and novel object phase (Contrast: 95% CI [0.05–0.41]). There were no significant differences between males and females in repeatability (see Figure S4).

#### 3.3.3 Aversive learning

Aversive learning was significantly repeatable in treatment zebrafish (*R* = 0.39, 95% CI [0.15–0.53]) but not in control group zebrafish (*R* = 0.12, 95% CI [0–0.31]; Figure 6c). When data were subset by sex, repeatability was insignificant in the control group for both males and females (males: *R* = 0.257, 95% CI [0–0.5]; females *R* = 0, 95% CI [0–0.3]; Figure S5), but significant in obesogenic treatment group zebrafish (males: *R* = 0.33, 95% CI [0.05–0.56]; females *R* = 0.46, 95% CI [0.20–0.66]; Figure S5). There was no significant difference in repeatability between control and treatment groups (Figure 6c) and between males and females.

### 3.4 Behavioral traits: comparing between‐individual correlations of personality traits

Overall, zebrafish who were bolder (spending more time close to the predator stimulus) also tended to be more neophilic (spending more time close to the novel object; boldness‐neophilia syndrome mean [95% credible intervals, CI]: 0.89 [0.66–1] Figure 7a). This was also reflected in both control and treatment groups (boldness‐neophilia syndrome mean [95% credible intervals, CI]: control: 0.66 [0.20, 0.96]; treatment: 0.88 [0.57, 1]; Figure 7b). There was also a trend for zebrafish who were more aggressive (spending more time near the single zebrafish stimulus) to be more social (spending more time near the shoal stimulus; aggression‐sociability syndrome mean [95% CI]: 0.75 [0.42–0.98]). However, analyzing control and obesogenic treatment groups separately, this behavioral syndrome was only statistically significant for the obesogenic treatment group (aggression‐sociability syndrome mean [95% CI]: control: 0.28 [−0.85, 0.96]; treatment: 0.78 [0.38, 0.99]). No other pair of behaviors produced a statistically significant between‐individual correlation (Figure 7a), and we found no significant contrasts in correlations among personality traits between control and obesogenic treatment groups (Figure 7b).

## 4 DISCUSSION

We have investigated the effects of an obesogenic diet on phenotype, focusing on behavior and cognition in zebrafish. To do this, we compared the phenotypes of zebrafish fed a control or obesogenic diet, with a multi‐faceted approach looking at differences in means, variability, repeatability (i.e., between‐individual consistencies in behavior) and behavioral syndromes (i.e., between‐individual level correlation between behavioral traits). We found zebrafish on an obesogenic diet were significantly heavier, displayed significantly more variation in body weight, but had similar levels of FBG to their control counterparts. In terms of behavior, treatment zebrafish spent significantly less time at the bottom of the tank (i.e., displayed more exploratory behavior) during the anxiety tank test and were less reactive to video stimuli with conspecifics during the personality test. However, these behavioral changes were highly sex‐specific with males spending more time in the bottom portion of the tank and being less reactive to social stimuli (Figures 3b and 4a,b). Zebrafish consuming an obesogenic diet also showed more variation in behavioral responses for these two assays than control zebrafish. Zebrafish responded similarly to aversive learning tests although the obesogenic diet group were significantly repeatable while the control zebrafish were not. However, there were no clear differences in repeatability between the two groups in most traits. In addition, while we found behavioral syndromes (significant correlations) between neophilia and boldness, and between sociability and aggression, these correlations did not differ between control and obesogenic groups of zebrafish. We discuss these main findings and additional insights in detail below.

### 4.1 The effect of obesogenic diet on weight and glucose levels

Zebrafish on an obesogenic diet (overfeeding of Artemia) were significantly heavier than their control counterparts in accordance with previous work (Hiramitsu et al., 2014; Landgraf et al., 2017; Oka et al., 2010; Tainaka et al., 2011). Although we did not explore more traits associated with increased body weight. Future studies are encouraged to delve into potential physiological disturbances to better understand how overfeeding impacts zebrafish. For example, previous studies have shown overfed zebrafish displayed elevated levels of triglycerides and fatty liver disease (Hiramitsu et al., 2014; Landgraf et al., 2017; Oka et al., 2010; Tainaka et al., 2011). Notably, body weight was significantly more variable in the overfed zebrafish. There are two main reasons for the latter result: (1) a well‐known positive mean and variance‐relationship (i.e., a higher mean of a trait results in a higher variance or standard deviation of the trait; [Cohen & Xu, 2015]), and (2) the obesogenic diet acting as a stressor, instigating more phenotypic variation (Chevin & Hoffmann, 2017). Many experiments have shown that organisms tend to have higher trait variances in stressful or new conditions (Hoffmann & Merilä, 1999). However, we found little impact of diet on mean levels of FBG. Previous studies in zebrafish models of diet‐induced obesity have also shown no impact on FBG levels. These zebrafish studies, however, did reveal subtle changes in glucose metabolism following meal administration and glucose tolerance tests (Carnovali et al., 2018; Hiramitsu et al., 2014). A limitation of our study is that we did not perform any kind of tolerance test. We suggest future studies employ more sensitive methods such as tolerance tests to detect impacts on blood glucose. Furthermore, tests to measure effects on other factors associated with obese phenotypes (e.g., triglycerides) will also assist in detecting physiological impairments.

### 4.2 The effect of obesogenic diet on anxiety‐related behavior

Zebrafish fed an obesogenic diet spent significantly less time in the low zone than control zebrafish during the anxiety tank tests (Figure 3b); that is, these zebrafish were less “anxious” and displayed more exploratory behavior, in contrast to a finding by Ghaddar et al. (2021). Rodent studies of diet‐induced obesity have also shown inconsistent results in exploratory behavior (Bracke et al., 2019; Zieba et al., 2019), although a recent meta‐analysis found obesogenic diets increased anxiety‐like behavior in elevated plus mazes as well as open field tests (Clark, Crean, & Senior, 2022). An increase in exploration is commonly associated with a “proactive” phenotype (Koolhaas et al., 2010; Øverli et al., 2007). Accordingly, zebrafish that spent more time in the lower portion of the tank can be seen as anxious as well as “reactive” (Koolhaas et al., 2010; Stewart et al., 2012). Importantly, this result was largely sex‐driven, with females from the treatment group spending less time in the low zone than males (Figure 3b). Indeed, female zebrafish have, in general, been shown to be less anxious (Genario, de Abreu, et al., 2020; Volgin et al., 2018), with some exceptions (dos Santos et al., 2021; Fontana et al., 2020; Genario, Giacomini, et al., 2020). Thus, subtle differences between males and females may only arise under certain contexts, as shown in a study by Marcon et al. (2022). Regardless, incorporating sex in studies examining nutritional effects on behavior will enable greater clarity in the nuanced interactions between diet and mood disorders such as anxiety (Clark, Reichelt, et al., 2022).

We speculate that morphology could have been a key factor moderating exploration behavior because treatment fish were significantly larger, particularly females due to sexual dimorphism in this species (Conradsen & McGuigan, 2015; Kern et al., 2016). For instance, the positive relationship between body size and boldness (an aspect of proactiveness) has been well documented in various species of fish (Brown & Braithwaite, 2004; Brown, Jones, & Braithwaite, 2007; Harris et al., 2010; Meuthen et al., 2019). Furthermore, it is also possible that patterns of feeding (e.g., frequency or schedule) rather than diet per se could influence exploration in zebrafish (Holley et al., 2014; Le Roy et al., 2021).

Both anxiety parameters – total distance traveled and time spent in the low zone – were also significantly repeatable (Figure 6a), in line with our previous work (Anwer et al., 2021). While not statistically different, control zebrafish tended to display overall higher repeatability estimates than those consuming an obesogenic diet, consistent with previous work by Baker et al. (2018). Zebrafish on an obesogenic diet also displayed more variation in time spent in the low zone, supporting our aforementioned explanation that an obesogenic diet generates more phenotypic variation (Fusco & Minelli, 2010).

### 4.3 Obesogenic diet affects personality traits and behavioral syndromes

Obesogenic zebrafish spent significantly less time close to video stimuli of conspecifics. This was the case for both the social phase and the aggression phase in the personality assay (Figure 4a,b). The former shows a school of fish while the latter shows a single fish; a significant correlation (behavioral syndrome) was observed between these two phases (Figure 7a). Therefore, we concluded that obese zebrafish were less likely to interact with conspecifics than control zebrafish. This effect was more pronounced in obese males than females. Given that nutritional content affects behavior, and that the balance of nutrients for optimal performance and fitness is usually sex‐specific, an obesogenic diet may have resulted in an imbalance which had greater influence on male social behavior (Han & Dingemanse, 2015; Reddiex et al., 2013). We did not observe any treatment or sex effects in the other types of behavioral responses: neophobia, boldness (Figure 4d), and activity level (Figure 4e). Our results of obese fish interacting less with conspecifics are in line with another zebrafish study (Audira et al., 2018). However, Picolo et al. (2021) found a short‐term high‐fat diet did not significantly change social preferences. Similarly, the rodent literature reports mixed evidence for the impact of obesogenic diets on social behavior (e.g., Reichelt et al., 2019, 2020; Takase et al., 2016). Thus, it is difficult to predict how diet would impact social behavior in zebrafish in a way that affects sexes differently.

Behavior of zebrafish consuming the obesogenic diet was significantly more repeatable during the aggression phase compared to the control group (Figure 6b). Aggression, or response to a conspecific, have been previously reported as repeatable traits in zebrafish (Fangmeier et al., 2018; Way et al., 2015). Therefore, while there were no statistically significant mean differences, the obesogenic diet seemingly impacted how consistent certain individuals were in response to video stimuli, driving repeatability estimates upwards. On the other hand, behavioral responses of control zebrafish were significantly more repeatable during the novel phase (Figure 7b). This seems to align with the repeatability results from our anxiety assay (i.e., control zebrafish displayed more consistent responses to a novel environment; Figure 6a). Additionally, we found a significant correlation between zebrafish responses during the novel and predator phases (Figure 7a). This is unsurprising considering response to predation (or threat) and response to novel objects are both associated with boldness, particularly in fish (Brown, Burgess, & Braithwaite, 2007; Thomson et al., 2012; Toms et al., 2010; Wilson & Stevens, 2005). This correlation, however, was not moderated by diet (Figure 7b) in our study. Also, the personality assay seemed to complement the results of anxiety assay, showing total distance traveled was unaffected by diet.

### 4.4 The effect of obesogenic diet on aversive learning

While obesogenic treatment and control zebrafish did not significantly differ in performance during the aversive learning assays (Figure 5). This result appears to contradict earlier zebrafish studies which have shown that high‐fat diets significantly impair the cognitive ability in a similar aversive learning assays (i.e., active avoidance tests; Meguro et al., 2019; Türkoğlu et al., 2022). High‐fat diets have also been shown to adversely affect cognition in other animal models such as rodents (Abbott et al., 2019; Beilharz et al., 2016; Jurdak et al., 2008; Kendig et al., 2019; Leigh et al., 2020). Furthermore, high‐fat diets impair learning abilities in humans, with several epidemiological studies showing that high‐fat/high‐energy intake is associated with poor cognition (Parrott & Greenwood, 2007; Yeomans, 2017).

Our seemingly contradictory results may stem from our overfeeding method (cf. Spencer et al., 2017); that is, we could not control nor quantify how much each individual was consuming within a tank. Therefore, fish were likely to have had variable consumption rates, influencing the cognitive impacts of overfeeding (Karoglu‐Eravsar et al., 2021; Kendig et al., 2019). Interestingly, repeatability of aversive learning was only significant in the treatment group, with some treatment zebrafish performing consistently poorer than control zebrafish (Figure 6c). Our previous work (Mason et al., 2021) showed repeatability of zebrafish on a normal diet is very low, matching the results of the current study and a meta‐analysis by Cauchoix et al. (2018). The low behavioral repeatabilities in the control fish indicates no individuals are consistently performing poorly. Taken together, our results suggest that an obesogenic diet does have an adverse effect in at least some of the treatment fish, however this did not translate into a significant difference between the treatment and control groups, possibly because our feeding regime did not impact all individuals equally.

## 5 CONCLUSION AND FUTURE DIRECTIONS

Our study used a multi‐faceted approach to examine the effects of an obesogenic diet on aspects of behavior and cognition in zebrafish. Although, we did not explore more physiological perturbances commonly associated with overfeeding, we found that zebrafish on an obesogenic diet displayed increased variation in several traits, exhibited more exploratory behavior during anxiety assays, and interacted less with video stimuli of conspecifics. Furthermore, these results were highly sex‐specific. An obesogenic diet also seemed to result in expression of more consistent behavioral responses in zebrafish across assays (i.e., repeatability). We hope our work and approach inspires a new generation of studies examining phenotypes in a more integrative and holistic manner, not only in zebrafish, but other animal models.

## AUTHOR CONTRIBUTIONS

**Hamza Anwer:** Conceptualization (lead); data curation (lead); formal analysis (lead); investigation (lead); methodology (lead); visualization (lead); writing – original draft (lead); writing – review and editing (lead). **Rose Eleanor O'Dea:** Data curation (supporting); formal analysis (supporting). **Dominic Mason:** Investigation (supporting); writing – review and editing (supporting). **Susanne Zajitschek:** Supervision (supporting); writing – review and editing (supporting). **Annabell Klinke:** Investigation (supporting); writing – review and editing (supporting). **Madeleine Reid:** Investigation (supporting); writing – review and editing (supporting). **Daniel Hesselson:** Resources (lead); writing – review and editing (supporting). **Daniel Noble:** Conceptualization (equal); methodology (equal); writing – review and editing (supporting). **Margaret Morris:** Supervision (lead); writing – review and editing (supporting). **Malgorzata Lagisz:** Data curation (supporting); supervision (lead); writing – review and editing (lead). **Shinichi Nakagawa:** Conceptualization (equal); funding acquisition (equal); methodology (equal); resources (lead); software (equal); supervision (lead); writing – review and editing (equal).

## ACKNOWLEDGEMENT

We are grateful for the staff at the Biological Testing Facility, Garvan Institute of Medical Research (in particular, to Miki Jahn) for their support and husbandry of zebrafish.

## FUNDING INFORMATION

This research was funded through an Australian Research Council Discovery grant (DP180100818) awarded to S. Nakagawa.

## Supporting information


Appendix S1

## Data Availability

All data and code can be accessed at the GitHub link: https://github.com/Apex619/Zebrafish‐obesity‐average‐variability‐repeatability‐and‐behavioural‐syndromes‐.
