# Supplementary material for: The effects of an obesogenic diet on behavior and cognition in zebrafish (Danio rerio): Trait average, variability, repeatability, and behavioral syndromes
Source: Ecol Evol. 2022 Nov 15;12(11):e9511. doi: 10.1002/ece3.9511 (PMC9666915; doi:10.1002/ece3.9511)
Supplement: Supplementary file 1 — Appendix S1 [file ECE3-12-e9511-s001.docx]

## The effects of an obesogenic diet on behaviour and cognition in zebrafish (Danio rerio): trait average, variability, repeatability, and behavioural syndromes

## Supplementary methods

**Creating experimental cohorts**

We set up 60 breeding pairs, over two days, from a stock of over 200 wildtype zebrafish. The wild-type stock was derived from of a mixture of Tübingen long fin, AB and other unidentified strains, which had been interbred for 8–10 generations to increase genetic diversity. We kept offspring from the 24 highest quality clutches to produce a total of 24 families. After 5 dpf, we transferred fry into independent nursery tanks upon which they were fed a standard facility diet of *Paramecium* twice daily up until 10-12 dpf when they were weaned onto live *Artemia* (twice a day) and dried fish food (once a day).

**Personality assay**

Experimental tanks and filming equipment were set up according to Fangmeier *et al.* (2018). Each tank was lined with two dark green acrylic panels to help minimize anxiety induced by novel surroundings (Blaser, Chadwick, and McGinnis 2010). Tanks were designed with a white floor to increase subject contrast during data extraction, and transparent acrylic short walls for presenting video stimuli via computer tablets. Tanks were positioned on temperature-controlled heat mats and set up in a quadrant formation, with a video camcorder positioned centrally overhead per four tanks. This setup was duplicated to film eight tanks in total simultaneously, with the two groups (four tanks in each) separated via a sheet of white corflute board. The entire experimental setup was positioned on a custom-made filming rig and surrounded by white corflute board to achieve uniform lighting and minimize visual disturbances during the filming process. Each experimental tank was filled with system water to 7cm depth and maintained at 28°C throughout the trials.

Each tank was set up with two computer tablets: one ‘control’ (tablet that continuously presented a neutral background image (blank, black side bars in Figure S1) and one ‘stimulus’ tablet that presented one of the 24 stimulus videos (red side bars in Figure S1). Blank tablets were always positioned on the outer ends of the experimental tanks, with stimulus tablets on the inner end (see Figure S1).

**Aversive learning assay**

Our experimental design involved assigning fish into quartets (that is, four fish that underwent a trial within a Zantiks unit simultaneously). These quartets were rotated systematically between trials to control for the time of day, Zantiks unit and lane position. Zantiks boxes were fully networked and controlled via a web browser. We opted for a blue colour unbiased conditioned (CS) and green colour unconditioned stimulus (US). Zebrafish were individually placed into one of four lanes of the Zantiks tank (20cm length x 14cm width x 15cm height; filled to 2.6L; see Figure S2). The assay consisted of 4 stages: acclimation, baseline, conditioning, and probe. Fish were acclimated for 30 minutes, whereby no stimuli was presented (neutral base). Following acclimation, half of the arena base displayed blue, and the other half green. Colours alternated every 5 minutes for 30 minutes to assess baseline preferences. The conditioning phase followed and consisted of the entire base displaying the CS (blue) for 1.5 seconds before delivering a mild electric shock (7V, 70ms). The entire base then displayed the US (green) for 8.5 seconds. This process was repeated 9 times. The final stage involved displaying both CS and US on different halves of the base (alternating every minute for 5 minutes; the probe period). One trial was approximately 70mins. Fresh system water was used for every trial.

## Supplementary figures


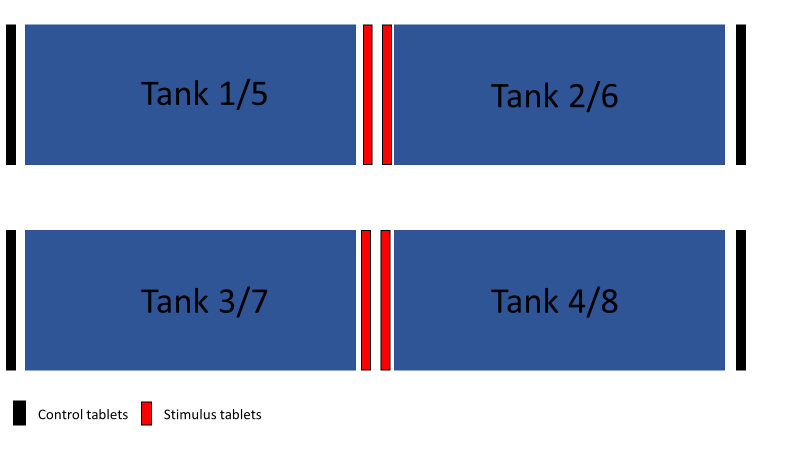


**Figure S1)** Experimental tanks were set up in a quadrant formation with cameras directly overhead (tank group 1 consisted of tanks 1–4, tank group 2 consisted of tanks 5–8). Blank tablets were positioned against the outer ends of the tanks while stimulus tablets were positioned against the inner ends of the tanks.


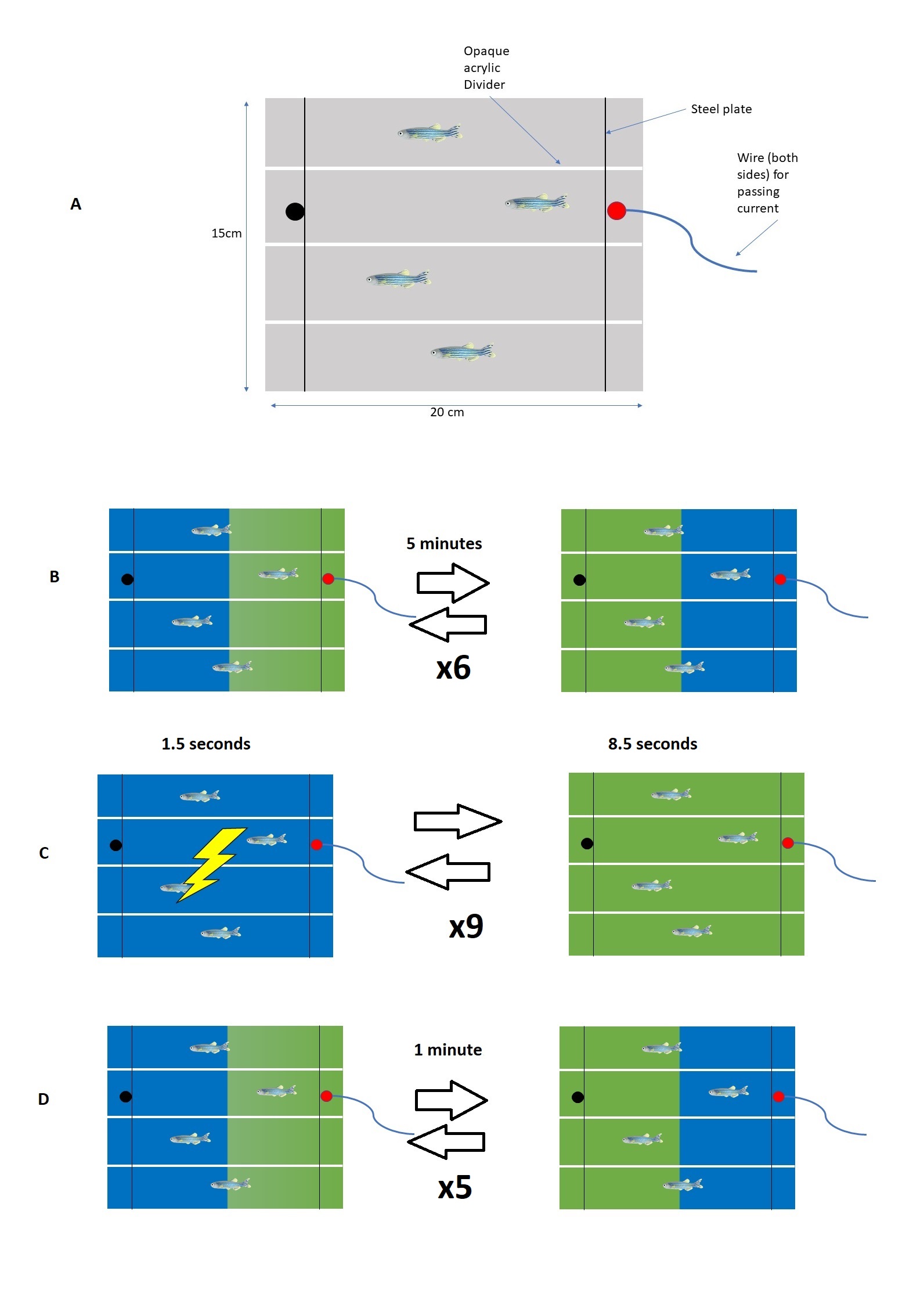


**Figure S2)** A) Aerial view of learning setup in the Zantiks tank. A blank base was displayed during the neutral acclimation period for 30 minutes B) Display during the baseline period (colours alternate sides every 5 minutes for 30 mins) C) The conditioning period; the conditioned stimuli (blue) is flashed for 1.5secs before a mild electric shock (7V, 70ms) is delivered. The base then switches to the unconditioned stimuli for 8.5 seconds D) Final stage where learning is assessed by repeating baseline protocol for 10 mins, with colours alternating sides every minute. Adapted from (Mason et al. 2021).


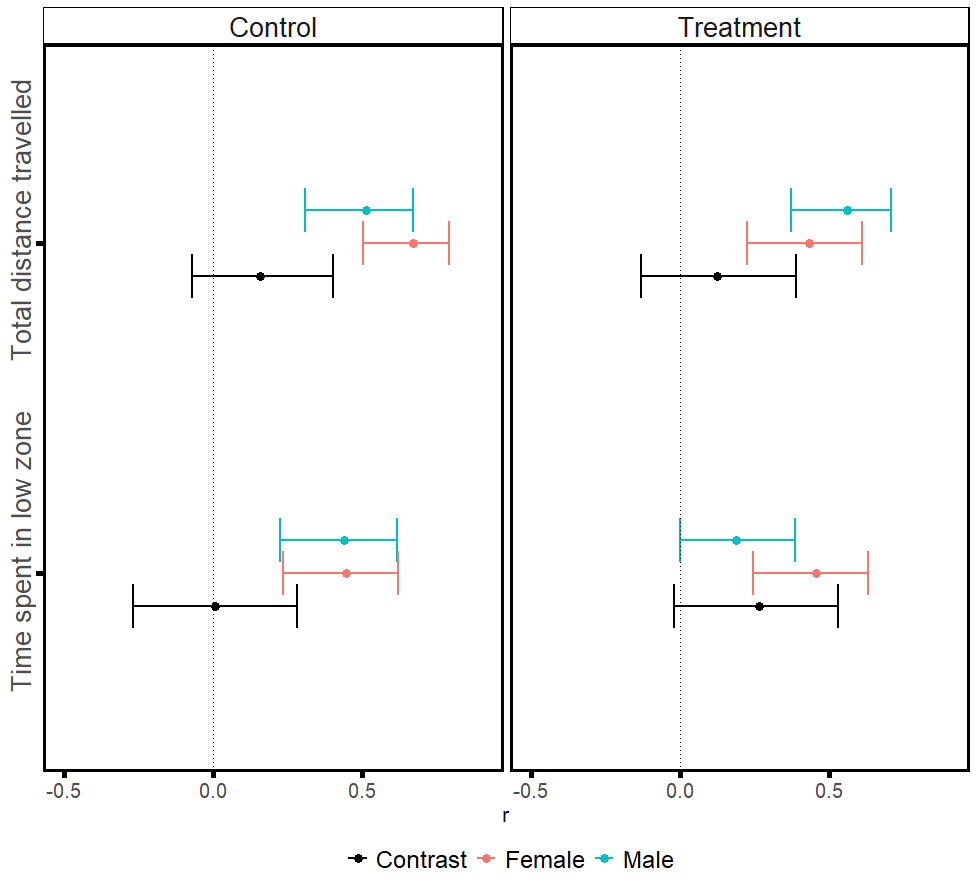


**Figure S3)** Forest plot of repeatability estimates for the behavioural parameters total distance travelled and time spent in the low zone. Estimates are displayed for both control and treatment tanks for males (blue) and females (red), as well as their contrast (in black). Repeatability estimates are deemed significant if the associated 95 % confidence interval does not cross 0. The contrasts are deemed significant if the associated confidence interval does not cross 0.


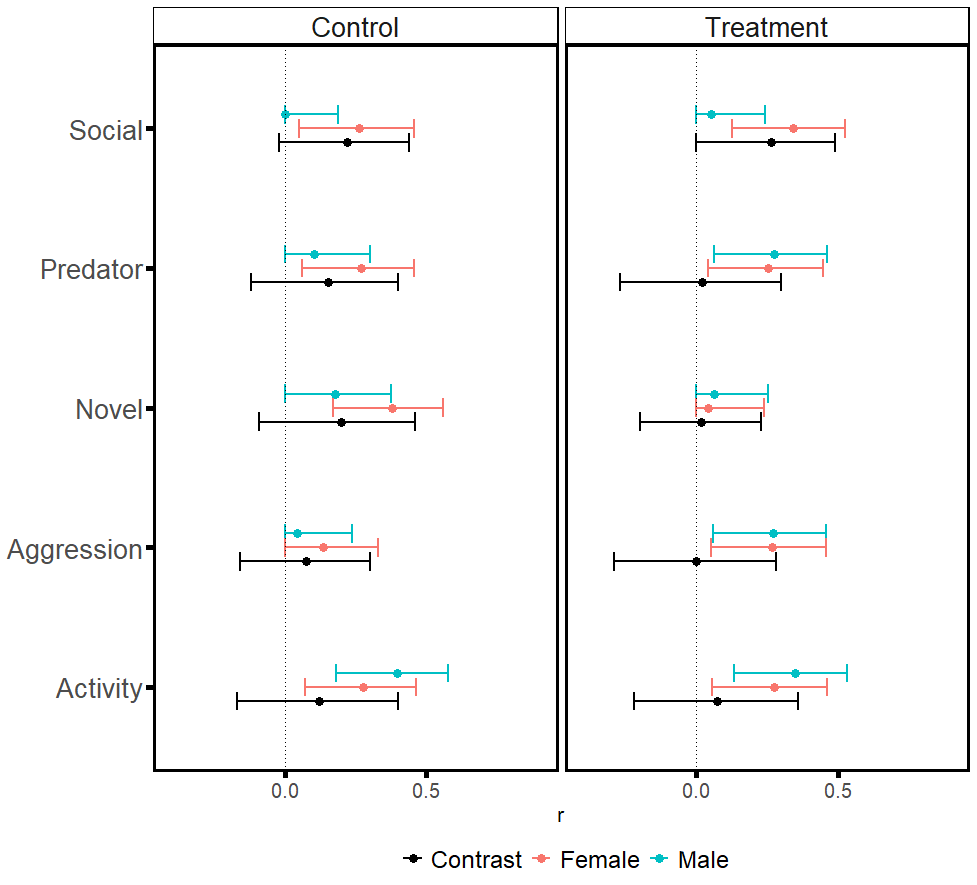


**Figure S4)** Forest plots repeatability estimates for personality traits of time spent near the stimulus during the social, predator, novel, and aggression phases, and total distance travelled for the activity phase; for both control and treatment tank males (blue) and females (red). Repeatability estimates are deemed significant if the associated 95 % confidence interval does not cross 0. The contrasts are deemed significant if the associated confidence interval does not cross 0.


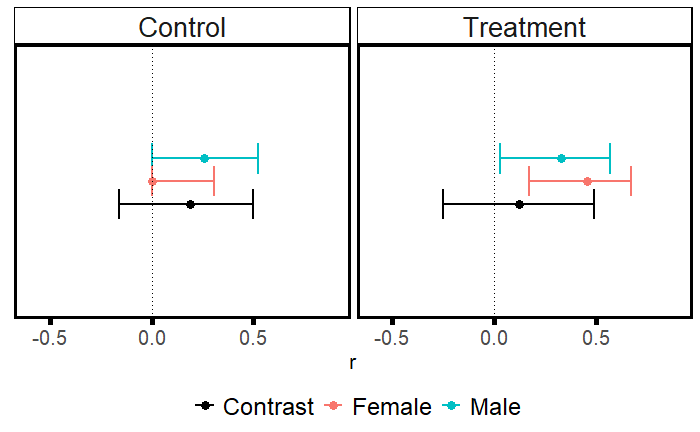


**Figure S5)** Forest plots repeatability estimates for aversive learning differences for both control and treatment tank males (blue) and females (red). Repeatability estimates are deemed significant if the associated 95 % confidence interval does not cross 0. The contrasts are deemed significant if the associated confidence interval does not cross 0.

## Supplementary tables

**Table S1*)*** Mixed model results for fasting blood glucose (FBG). Contrasts are provided for sex differences, group differences and interaction differences. Point estimates (means) are provided as well as 95% confidence intervals (CI) and p-values. Statistically significant results are highlighted in bold.

|  | **FBG** | | |
| --- | --- | --- | --- |
| *Predictors* | *Estimates* | *CI* | *p* |
| (Intercept) | 3.34 | 3.00 – 3.69 | **<0.001** |
| Sex [male] | -0.76 | -1.25 – -0.27 | **0.003** |
| Group [Treatment] | -0.04 | -0.54 – 0.45 | 0.861 |
| Sex [male] * Group [Treatment] | 0.23 | -0.46 – 0.92 | 0.505 |

**Table S2*)*** Mixed model results for total distance travelled. Contrasts are provided for sex differences, group differences and water condition. Point estimates (means) are provided as well as 95% confidence intervals (CI) and p-values. Statistically significant results are highlighted in bold

|  | **Total distance travelled** | | |
| --- | --- | --- | --- |
| *Predictors* | *Estimates* | *CI* | *p* |
| (Intercept) | 986.23 | 819.16 – 1153.30 | **<0.001** |
| Group [Treatment] | 210.33 | -29.96 – 450.62 | 0.086 |
| Sex [male] | 209.67 | -24.02 – 443.35 | 0.078 |
| Water_ID | -25.09 | -48.17 – -2.01 | **0.033** |
| Group [Treatment] * Sex [male] | -184.52 | -519.56 – 150.52 | 0.278 |

## References

Blaser, R E, L Chadwick, and G C McGinnis. 2010. “Behavioral Measures of Anxiety in Zebrafish (Danio Rerio).” *Behavioural brain research* 208(1): 56–62.

Fangmeier, Melissa L et al. 2018. “Computer Animation Technology in Behavioral Sciences: A Sequential, Automatic, and High-Throughput Approach to Quantifying Personality in Zebrafish (Danio Rerio).” *Zebrafish* 15(2): 206–10.

Mason, Dominic et al. 2021. “ Low Repeatability of Aversive Learning in Zebrafish ( Danio Rerio ) .” *Journal of Experimental Biology* 224(11). https://osf.io/t95v3/ (June 16, 2021).
